# Xanthogranulomatous Pyelonephritis in a Transplant Kidney Leading to Severe Allograft Dysfunction

**DOI:** 10.1155/crit/6605652

**Published:** 2025-02-21

**Authors:** Usman Baig, Ahmad Mirza, Payaswini Vasanth, Laura Mulloy, Shameem Beigh, Imran Gani

**Affiliations:** ^1^Department of Nephrology, Hypertension and Transplant Medicine, Wellstar MCG Health, Augusta University, Augusta, Georgia, USA; ^2^Department of Surgery, Wellstar MCG Health, Augusta University, Augusta, Georgia, USA; ^3^Department of Nephrology, Emory University, Atlanta, Georgia, USA

**Keywords:** kidney transplantation, renal allograft dysfunction, urinary tract infection, xanthogranulomatous pyelonephritis

## Abstract

Xanthogranulomatous pyelonephritis (XPN) is a rare and unusual form of pyelonephritis that infrequently occurs in renal allografts. Clinical presentation ranges from asymptomatic to fever, pyuria, and graft dysfunction. We present a case of a young African American male who presented with a marked elevation in serum creatinine from a baseline of 1.8–1.9 to 9.86 mg/dL. Transplant kidney biopsy was consistent with the diagnosis of XPN, showing xanthoma cells, which are pathognomonic for this condition. Following antibiotic treatment, allograft function improved and return to dialysis was averted. Clinicians should consider XPN in transplant patients with deteriorating allograft function, as its presentation can mimic more common conditions. Graft salvage with appropriate antimicrobial therapy should be attempted before considering nephrectomy which risks reinitiation of dialysis.

## 1. Introduction

Although acute allograft pyelonephritis is common in kidney transplant patients, xanthogranulomatous pyelonephritis (XPN) is an aggressive type of pyelonephritis that rarely occurs in kidney allografts. It is characterized by renal parenchymal necrosis, in which the damaged tissue is replaced by xanthoma cells (macrophages filled with lipids). In the general population, it has an incidence of 1.4 cases per 100,000 individuals in the native kidneys [1]. It is more common in females, with a ratio of 2:1 [2]. It typically occurs after the fourth decade of life, often with a history of repeated urinary tract infections (UTIs) or nephrolithiasis [3]. The complication rate for XPN ranges from 30% to 40%. The most frequent complications are sepsis, spread to contiguous structures, and risks associated with nephrectomy [4, 5].

We present a case of a young African American male who, 8 months after receiving a renal transplant, presented with marked elevation of creatinine due to XPN, with an overview of his management and literature review. Our case is unique given the patient's minimal clinical symptoms, significantly elevated creatinine that was concerning for allograft rejection, and normal ultrasound imaging.

## 2. Case Report

A 30-year-old African American male, with past medical history of hypertension, hyperlipidemia, and end-stage renal disease secondary to poststreptococcal glomerulonephritis, received a 6-antigen mismatch deceased donor kidney transplant in September 2023. He made an uneventful recovery after the transplant surgery. Posttransplant follow-up appointments were as per our transplant center protocol, and his baseline line creatinine settled between 1.8 and 1.9 mg/dL. In July 2024, he presented to the outpatient transplant clinic for a routine visit. For the last 7 days, he had noticed foul-smelling and cloudy urine. He did not seek medical attention. He felt fine overall and denied dysuria, hematuria, fever, or chills. He self-medicated with cranberry supplements, vitamin C, and phenazopyridine (over-the-counter urinary analgesic). His immunosuppressant medication history included extended-release tacrolimus (10 mg, daily), mycophenolate mofetil (500 mg, twice daily), and prednisone (5 mg, daily). His vitals were stable: heart rate of 90 bpm, respiratory rate of 14 per minute, blood pressure of 125/80 mmHg, and blood oxygen saturation of 100%. Physical examination was unremarkable. His serum creatinine was 9.86 mg/dL, blood urea nitrogen was 125 mg/dL, serum potassium was 3.5 mEq/L, and serum bicarbonate was 19 mEq/L. He was not uremic. He gave history of missing a few doses of mycophenolate mofetil. Tacrolimus level was slightly elevated at 11.3 ng/mL (than his goal level of 6–8 ng/mL) for which tacrolimus dose adjustments were made. Given substantially deranged renal function and missed immunosuppressant doses (mycophenolate mofetil), he was admitted to hospital for suspicion of transplant rejection.

Total leucocyte count was normal (9700/microliter) at presentation. Urinalysis showed cloudy urine, bacteriuria, leukocyturia, and hematuria. It also was positive for proteinuria of 50 mg/dL, nitrites, and leukocyte esterase. Clinical history and a normal prostate-specific antigen level of 0.06 ng/dL did not point toward acute prostatitis. Ultrasound of the transplanted kidney showed no nephrolithiasis, hydronephrosis, or renal artery stenosis. Donor-specific antibodies (DSAs) were negative. BK virus PCR in plasma was negative. Blood culture was negative as well. Empiric ceftriaxone (1000 mg IV, once daily) was initiated for the treatment of the UTI following the institutional antibiogram and in line with the acceptable first-line treatment. The patient underwent an allograft biopsy due to strikingly elevated creatinine. Later, his urine culture grew pan-sensitive *Escherichia coli* (including sensitivity to ceftriaxone).

**Figure 1:**
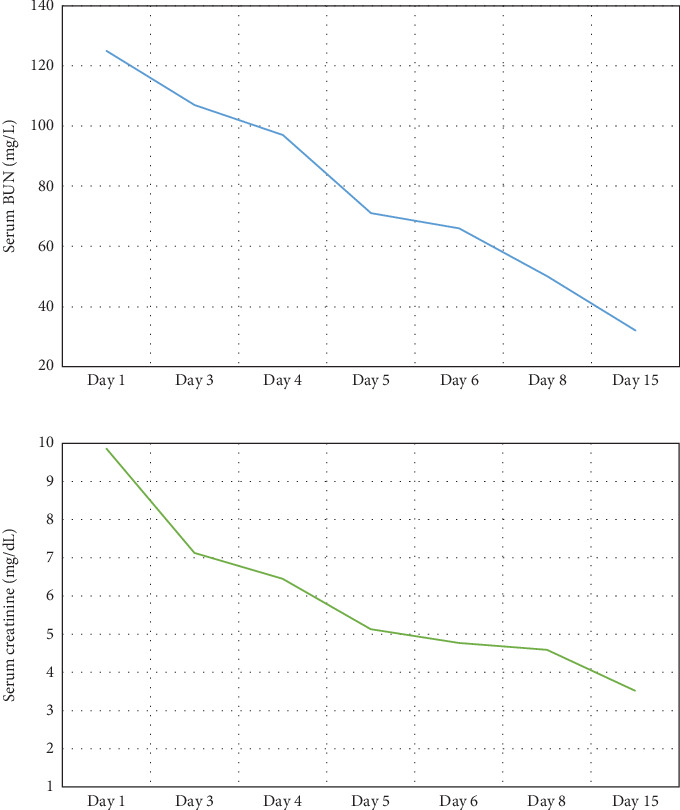
Decreasing trends of serum creatinine and BUN over the course of treatment.

**Figure 2:**
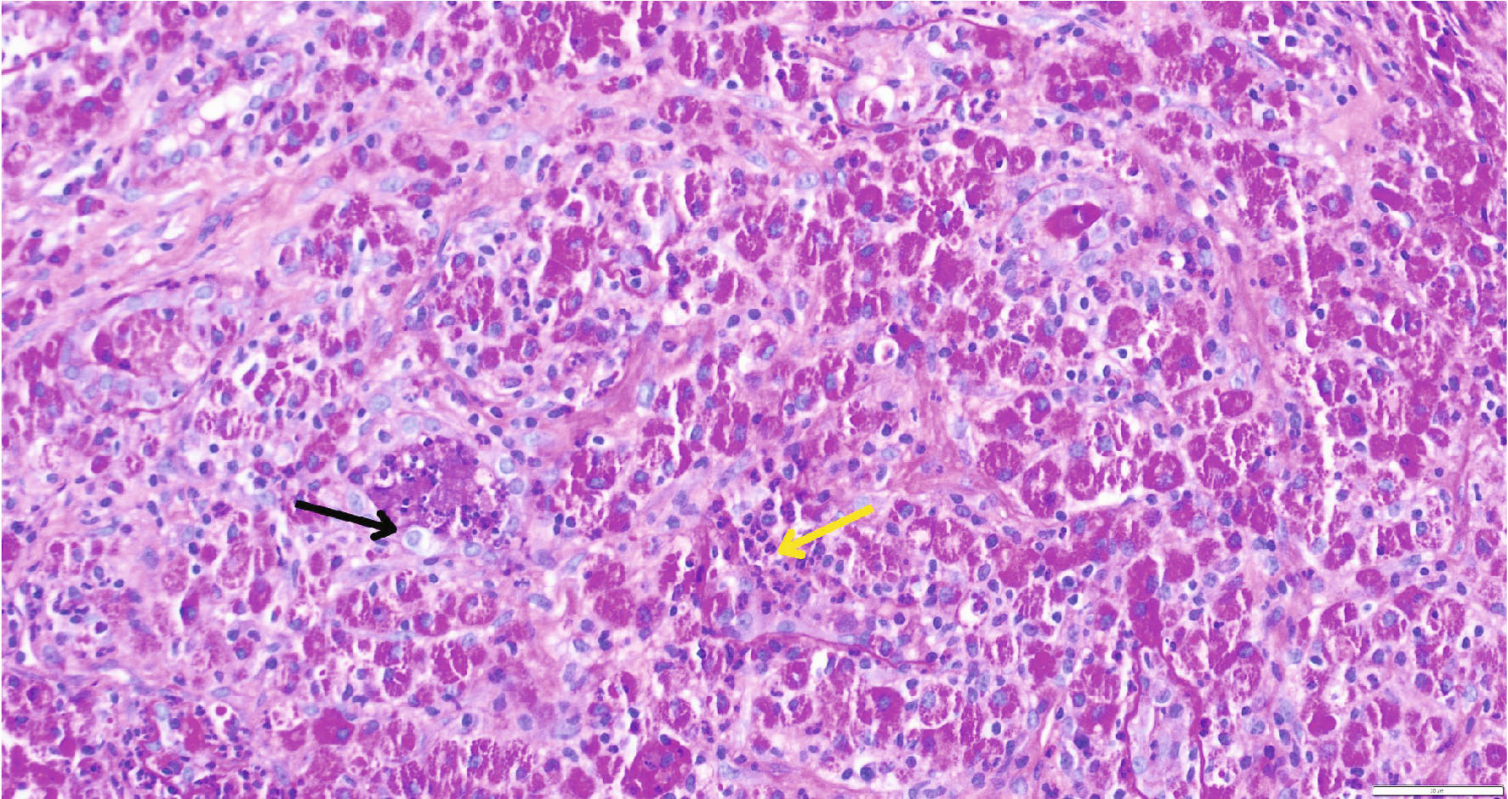
Xanthogranulomatous inflammation is shown. Yellow arrow indicates inflammatory cells, while black arrow points to lipid-laden macrophages (xanthoma cells).

**Figure 3:**
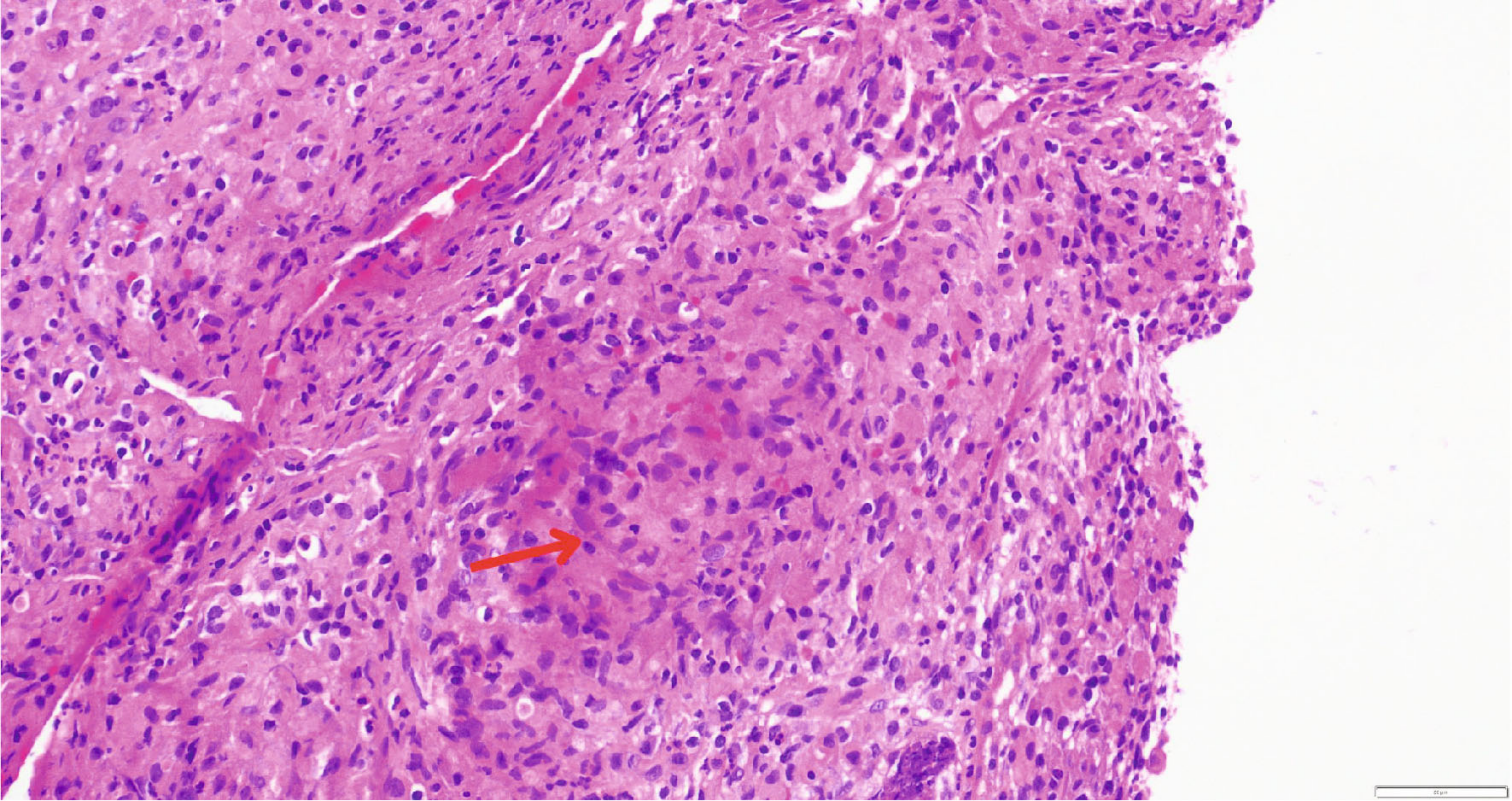
Necrotizing granuloma, marked by red arrow.

**Figure 4:**
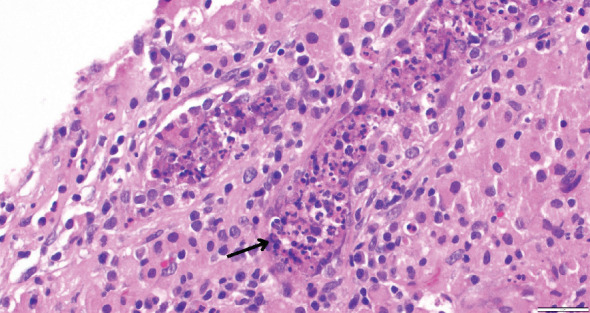
Neutrophilic cast within a tubule, marked by black arrow.

**Figure 5:**
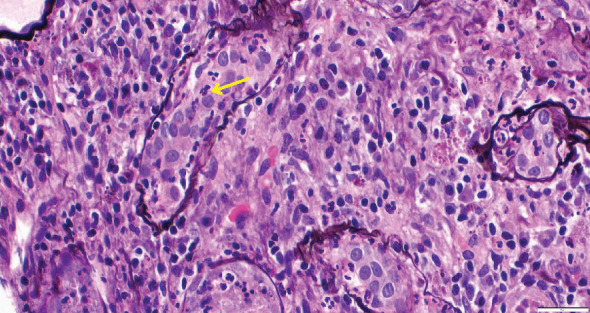
Neutrophilic tubulitis, with polymorphonuclear leukocytes (PMNs) marked by yellow arrow.

While on ceftriaxone, his creatinine decreased to 5 mg/dL and BUN to 70 mg/dL over the next 3 days (Figure 1). He did not need renal replacement therapy. Allograft biopsy showed dense tubulointerstitial inflammation with frequent neutrophils, plasma cells, and foamy histiocytes, findings diagnostic of XPN (Figure 2). Sheets of histiocytes distorted the renal parenchymal architecture. There was focal nonnecrotizing granulomatous inflammation, and numerous neutrophilic tubular casts were present (Figures 3 and 4). The tubular epithelium was severely attenuated, with neutrophilic tubulitis (Figure 5). There was no evidence of endothelialitis, glomerulitis, and peritubular capillaritis. C4d stain was negative. There was no mesangial expansion, subepithelial humps, arteriolar hyalinosis, fibrinoid necrosis, segmental sclerosis, or crescent formation. The SV40 immunohistochemical stain for BK/polyomavirus, the stains for CMV and adenovirus, and the Von Kossa stain for Michaelis–Gutmann bodies of malakoplakia were all negative. The sections were also stained for IgG, IgM, IgA, C3, and C1q, and they were negative in both the glomeruli and extraglomerular space.

Treatment with intravenous ceftriaxone was continued for 5 more days given the severity of infection and in an attempt to achieve complete eradication, thus reducing the chances of relapse in a single kidney model. Mycophenolate mofetil dose was decreased (to 250 mg, PO twice daily) for the duration of treatment. On Day 6, creatinine decreased to 4.5 mg/dL, and BUN decreased to 50 mg/dL. The patient was discharged on oral ciprofloxacin for 3 weeks. At outpatient follow-up 1 week later, his serum creatinine and BUN further decreased to 3.5 and 32 mg/dL, respectively. Repeat urinalysis was unremarkable. Over the next few months, he maintained his serum creatinine around 3.5 mg/dL.

## 3. Discussion

Allograft pyelonephritis involves bacterial infection of the transplanted kidney. It usually occurs secondary to the ascending infection from the bladder and urethra. The absence of a sphincter between the donor kidney ureter and the recipient bladder is a risk factor for transplant руеloneрhritis [6]. It can present with nonspecific symptoms like fever, malaise, chills, and nausea but may be associated with graft tenderness, foul smelling urine, and acute elevations in serum creatinine. Allograft pyelonephritis may or may not be preceded by symptoms of cystitis (dysuria, frequency, urgency, hematuria, and suprapubic pain). Associated laboratory abnormalities include positive leukocyte esterase on dipstick testing, leukocyturia, leukocytosis, and usually a positive urine culture. Bacteremia with the same organism as in urine can be seen [6, 7].

Although transplant pyelonephritis is common, transplant kidney XPN is extremely rare and may not be initially suspected. In 1916, Schlagenhaufer first described XPN, which was subsequently termed xanthogranuloma by Osterlin in 1944 [8]. XPN is a rare variant of pyelonephritis, typically seen in patients with repeated urinary tract obstruction and UTIs. Its pathophysiology most likely involves a defect in bacterial degradation by macrophages, leading to inflammation and damage to the renal parenchyma. The most common bacteria linked are *E. coli* and *Proteus mirabilis*, with *Pseudomonas aeruginosa* and *Enterococcus faecalis* being less frequently involved [2, 9]. In renal transplant recipients with XPN specifically, *E. coli* and *Klebsiella* species are the predominant bacteria isolated in cultures [10]. Urinary tract obstruction may be caused by staghorn calculus which serves as a nidus for infection. Other risk factors associated are metabolic syndrome, abnormal lipid metabolism, hypertension, hepatitis C, immunocompromised state, and renal transplantation [11].

The common presenting symptoms in native kidneys include fever, chills, flank pain, hematuria, dysuria, and urinary urgency. Physical examination may show a palpable flank mass and costovertebral tenderness. Extrarenal manifestations may include the spread of infection to adjacent structures [12].

However, in a transplant patient, typical signs and symptoms are lacking due to immunosuppression and denervated transplant kidney. Diagnosis is confirmed by renal biopsy. Allograft rejection was also higher on our differential due to severity of acute kidney failure and noncompliance with mycophenolate mofetil. Laboratory evaluation usually shows elevated blood leukocyte count. Inflammatory markers such as erythrocyte sedimentation rate (ESR) and C-reactive protein (CRP) are typically elevated. Findings consistent with UTI are seen on urinalysis. Urine cytology may show xanthoma cells in up to 30% of patients. While these cells are specific, they have low sensitivity and were not observed in our case [12, 13]. Imaging and biopsy help guide treatment and are essential for reaching a definitive diagnosis. In cases associated with staghorn calculus, abdominal x-ray shows radiopaque shadow at the ureteropelvic junction. Renal ultrasound usually shows an enlarged kidney, multiple hypoechoic shadows, and hydronephrosis [3]. CT scan is useful in determining the extent of involvement and classifying the stages of XPN [14]. CT scan was not obtained in our case due to grossly unremarkable transplant kidney ultrasound and aim to avoid contrast dye exposure. Proceeding with an allograft biopsy ultimately led to the diagnosis of XPN.

Malakoplakia, which typically occurs in immunocompromised individuals, should be ruled out when considering a diagnosis of XPN. It is a chronic inflammatory condition that can involve multiple systems and presents with histology consisting of mixed inflammatory cells along with foamy histiocytes with eccentric, hyperchromatic nuclei and eosinophilic granules. These features can resemble those seen in XPN. To differentiate, periodic acid-Schiff (PAS) stain, which is positive for Michaelis–Gutmann bodies, along with Perl's stain and Von Kossa stain, can be used [15, 16]. Our patient stained negative for Von Kossa stain for Michaelis–Gutmann bodies.

Treatment is initiated with empirical antibiotics, which usually include third-generation cephalosporins, extended-spectrum penicillins, or carbapenems [17]. We chose ceftriaxone for the empiric treatment. The antibiotic was later confirmed to be effective from the beginning, as the *E. coli* strain was pan-sensitive. The decision to switch from IV to oral antibiotics depends upon various factors including clinical improvement, ability to take oral antibiotics, hemodynamic stability, and culture results, as no established consensus exists. We transitioned to an oral fluoroquinolone (ciprofloxacin), which has high serum bioavailability and also achieves a high concentration in the urinary system. This approach proved effective, as no recurrence was observed.

In most nontransplant cases, where the predisposing factor is either recurrent UTI or staghorn calculus, antibiotics are rarely sufficient, and surgical resection is required. Nephron-sparing surgery (i.e., partial nephrectomy) can be done in a very small number of cases; however, in most cases, total nephrectomy is required. Nephrectomy approach used can be either open or laparoscopic, with recent evidence favoring the latter [17].

The prognosis of XPN is influenced by patient and allograft factors and response to antibiotics. Early detection and treatment are essential, as prompt management may preserve the graft and avoid reversion to renal replacement therapy.

## 4. Conclusion

XPN is a rare but aggressive form of pyelonephritis that infrequently occurs in renal transplant patients. Clinicians should remain vigilant for this diagnosis in cases of deteriorating kidney function, as its clinical presentation may mimic more common conditions. Diagnostic confirmation typically requires imaging and biopsy. Initiating appropriate antibiotic therapy based on culture and sensitivity is crucial in cases of XPN in allografts. Appropriate response when seen avoids nephrectomy and return to dialysis in this single kidney model patient population. Allograft function may not return to baseline.

## Data Availability

The relevant clinical data from this case report are included in the manuscript, with all data anonymized to the best possible extent.
